# Unrequested Findings on Cardiac Computed Tomography: Looking Beyond the Heart

**DOI:** 10.1371/journal.pone.0032184

**Published:** 2012-04-19

**Authors:** Constantinus F. Buckens, Helena M. Verkooijen, Martijn J. Gondrie, Pushpa Jairam, Willem P. Mali, Yolanda van der Graaf

**Affiliations:** 1 Department of Radiology, University Medical Center Utrecht, Utrecht, The Netherlands; 2 Julius Center for Health Sciences, University Medical Center Utrecht, Utrecht, The Netherlands; University Medical Center Rotterdam, The Netherlands

## Abstract

**Objectives:**

To determine the prevalence of clinically relevant unrequested extra-cardiac imaging findings on cardiac Computed Tomography (CT) and explanatory factors thereof.

**Methods:**

A systematic review of studies drawn from online electronic databases followed by meta-analysis with meta-regression was performed. The prevalence of clinically relevant unrequested findings and potentially explanatory variables were extracted (proportion of smokers, mean age of patients, use of full FOV, proportion of men, years since publication).

**Results:**

Nineteen radiological studies comprising 12922 patients met the inclusion criteria. The pooled prevalence of clinically relevant unrequested findings was 13% (95% confidence interval 9–18, range: 3–39%). The large differences in prevalence observed were not explained by the predefined (potentially explanatory) variables.

**Conclusions:**

Clinically relevant extra-cardiac findings are common in patients undergoing routine cardiac CT, and their prevalence differs substantially between studies. These differences may be due to unreported factors such as different definitions of clinical relevance and differences between populations. We present suggestions for basic reporting which may improve the interpretability and comparability of future research.

## Introduction

Improvements in the quality of cardiac Computed Tomography (CT) are driving its increasingly widespread use in an expanding patient-group [1]. These same improvements and the increased number of cardiac CT scans are also resulting in the increasing detection of unrequested findings. These unrequested (‘ancillary’ or ‘incidental’) findings are more frequently visible on advanced high-resolution scans but fall beyond the reasonable remit of the initial indication for imaging and thus beyond what has been explicitly requested by referring clinicians.

Whilst they apply to all diagnostic imaging modalities, unrequested findings are particularly germane to cardiac CT due to the density of organ systems in the chest and the practice of exclusively evaluating the cardiac/coronary structures. Furthermore, typical patients referred for cardiac CT may also be relatively prone to co-morbidities, due to the confluence of wide-ranging (cardiovascular) risk factors, such a smoking, hypertension, diabetes and obstructive pulmonary disease [2].

Concerns over the growth of healthcare consumption and radiation exposure are driving calls for the efficient use CT [3]–[6]. Preventing unnecessary follow-up stemming from irrelevant un-requested findings and systematically reporting on prognostically relevant imaging information could contribute to this. Unfortunately, there is little clarity about which (classes of) findings hold relevance and which do not, although this is beginning to be addressed [7].

This uncertainty poses a challenge to radiologists and referring physicians alike, with responses ranging from calculated disregard to evaluation of all imaging data available and aggressive follow-up of unrequested findings [8], [9]. Often the only rationale provided is expert opinion or prevailing tradition. The fact that these unrequested findings can be detected without additional radiation exposure is pitted against the indeterminate significance of many unrequested findings and the risk and cost of provoking unnecessary follow-up.

Here we review those publications examining the prevalence of incidental findings amongst patients referred for routine cardiac CT scans and assess the effect of candidate explanatory factors abstracted from these articles through a systematic search, review and meta-analysis with meta-regression.

## Materials and Methods

### Systematic Review: Search and Inclusion

A systematic review method was employed to ensure comprehensive coverage of the available evidence. The Meta-analysis of observational studies (MOOSE) checklist [10] was consulted during the writing of this article (Supplement S1). A systematic electronic search was performed on 15-09-2011 using the MEDline, EMBASE and Cochrane databases. Synonym lists were generated to describe our intended domain and outcome: adult patients undergoing routine cardiac CT and (overall) prevalence of unrequested findings. These were subsequently used to build the search (Table 1).

**Table 1:** Query syntax for MEDline, EMBASE, and the Cochrane Library.

Database	Search strategy
MEDline	((“computed tomography”[tiab] OR CT[tiab]) AND (thora*[tiab] OR chest[tiab] OR cardiac[tiab])) AND (incidental[tiab] OR accidental[tiab] OR ancillary[tiab] OR extra-coronary[tiab] OR non-coronary[tiab] OR extracardiac[tiab] OR extra-cardiac[tiab] OR non-cardiac[tiab])
EMBASE	‘computed tomography’:ab,ti OR ct:ab,ti AND (thora*:ab,ti OR chest:ab,ti OR cardiac:ab,ti) AND (incidental:ab,ti OR accidental:ab,ti OR ancillary:ab,ti OR ‘extra coronary’:ab,ti OR ‘non coronary’:ab,ti OR ‘extracardiac’:ab,ti OR ‘extra cardiac’:ab,ti OR ‘non cardiac’:ab,ti) AND [embase]/lim
The Cochrane Library	(((computed tomography):ti,ab,kw or (CT):ti,ab,kw) AND ((thora*):ti,ab,kw or (chest):ti,ab,kw or (cardiac):ti,ab,kw)) AND ((incidental):ti,ab,kw or (accidental):ti,ab,kw or (ancillary):ti,ab,kw or (extra-coronary):ti,ab,kw or (non-coronary):ti,ab,kw or (extracardiac):ti,ab,kw or (extra-cardiac):ti,ab,kw or (non-cardiac):ti,ab,kw)

**Figure 1:**
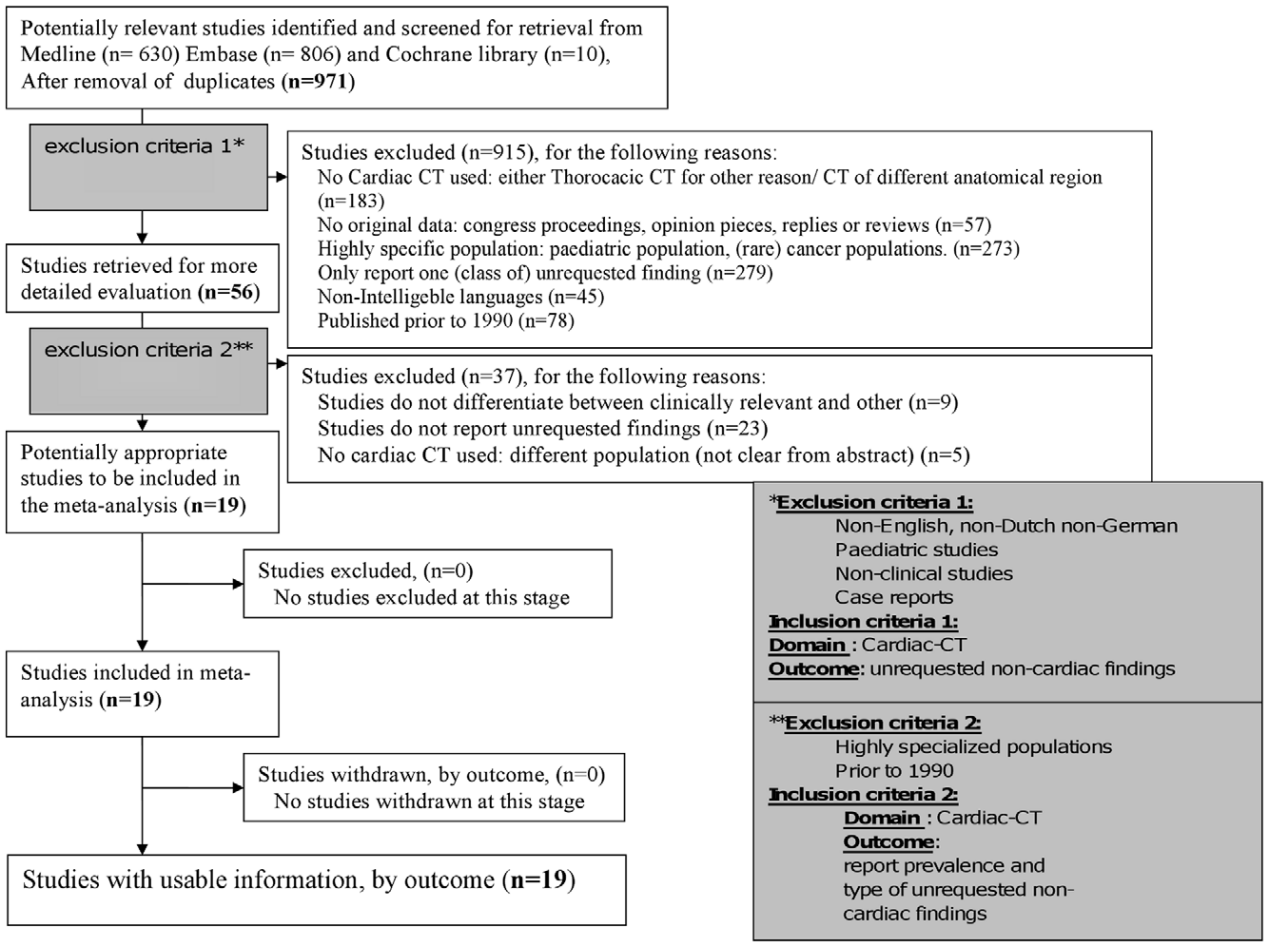
Flowchart illustrating literature search and selection procedure.

The titles and abstracts from the different databases resulting from this search were combined and duplicates were manually filtered. The remaining articles were then subjected to the selection procedure further outlined in Figure 1. Briefly, the titles and the abstracts were screened by two experienced medical researchers independently (CFB and MJAG) on the basis of predefined exclusion and inclusion criteria (exclusion and inclusion criteria 1, figure 1), largely to ensure general applicability of the articles. Briefly, we assessed whether the abstracts retrieved by the search reported on extra-cardiac findings on cardiac CTs met the inclusion criteria 1 (figure 1). Studies published before 1990 were excluded due to the non-comparability in access to and quality of CT-scanning between recent years and the 1980s. Full text papers meeting these criteria were screened using the second set of inclusion and exclusion criteria (exclusion and inclusion criteria 2, Figure 1). This second set of inclusion and exclusion criteria were intended to discriminate between articles containing truly useful information and those that were less relevant to routine clinical practice. This included studies investigating incidental CT findings in highly specialized subpopulations (e.g. only patients with cardiac tumours, patients with sarcoidosis), studies only reporting on one (class of) unrequested/incidental finding (e.g. breast lesions [11] or cardiac abnormalities [12]) and studies that turned out not to report on unrequested cardiac findings after full-text review.

### Systematic Review: Data Extraction

The ‘STrengthening the Reporting of OBservational studies in Epidemiology’ (STROBE) [13], [14] checklist for cross-sectional studies was used as a framework to assess the quality of the reporting in the included articles. We selected those items pertaining to the reporting of study population and (completeness of) reporting of results and adapted them so that they would more specifically address the prevalence of incidental findings on cardiac CTs.

Briefly, we deemed the items concerning the setting, the sources/eligibility criteria of participants, and the description of study participant characteristics and the reporting of results to be the most germane and these were further specified to our research question. The resulting specified items, alongside the original STROBE items from which they were derived, (Table 2) were scored as present or absent by two authors independently. The items were scored as present if the item was reported adequately anywhere in the assessed article. In the case of referral source, an item was scored as reported if it was clear how the study population came to be referred for cardiac scanning. For the prevalence of CVD risk factors, we required that the prevalence of the major risk factors (smoking, hypertension and some form of CVD history) be reported. Finally, we assessed whether the absolute numbers of unrequested findings as well as their prevalence could be delineated.

**Table 2:** Selected items from STROBE checklist, together with percentage and number of articles in which items were scored ‘yes’.

**STROBE item number**	**5**	**6(a)**	**14a**	**15**
**description of STROBE item (verbatim from STROBE** **checklist)**	Describe the setting, locations,and relevant dates, includingperiods of recruitment, exposure,follow-up, and data collection	Give the eligibility criteria,and the sources and methodsof selection of participants	Give characteristics of study participants (eg demographic, clinical, social) and information on exposures and potential confounders	Report numbers of outcome events or summary measures
**percentage of articles reporting**	**65**	**41**	**76**	**100**
**number of articles reporting**	**11**	**7**	13	7
**Corresponding specification of STROBE item**	Referral source (clarifies which portion of included patients areself-referral, from primary care, from emergency care, fromintramural specialist care, screening)		Reports the prevalence of CVD risk factors and comorbidities in included patients (eg smoking, hypertension, history of CVD)	results for individual (types of) findings given in absolute numbers as well as prevalences
**percentage of articles reporting**	**29**		35	12
**number of articles reporting**	**5**		6	2

Data on study parameters and the prevalence of unrequested findings were extracted from the included papers by two authors independently, with consensus sought in cases of disagreement. The primary outcome of interest was clinically relevant unrequested findings, defined as those unrequested findings which required short-term follow-up, either with further diagnostic procedures or therapeutic interventions.

### Meta-analysis and Meta-regression

All statistical analyses were carried out using the R statistical program [15] version 2.13.1. Meta-analysis and meta-regression were carried out using the metafor [16] package version 1.6.0. We pooled the reported prevalence’s of Clinically Relevant Unrequested findings in order to come to more meaningful conclusions (Table 2). By then assessing heterogeneity and performing univariate meta-regression we sought to assess the degree of ‘differentness’ and to then explain it using easily extracted study parameters, such as the age of the patient group. The proportions of clinically relevant unrequested findings were logit transformed to improve approximate normality. These were used in the analyses and meta-regression, with the results being back-transformed before presentation here. Heterogeneity was assessed by computing the proportion of unexplained variance using the I^2^ and Tau^2^ statistics [17]. Pooled estimates were generated using restricted maximum likelihood estimator random effect approach when the I^2^ was found to be higher than 25% [18]; this random effects approach makes allowances for the excess heterogeneity the I^2^ statistic reflects. Funnel plots were generated and visually inspected for approximate symmetry to assess the risk of publication bias.

For the meta-regression, mixed effects regression using unrestricted maximum likelihood estimator method was employed to estimate the effects of potentially explanatory variables that could be abstracted from the articles. The reported mean age, proportion of smokers, years since publication and use of full Field Of View (FOV; whether or not all available anatomical regions were assessed) were considered as potentially explanatory for differences in the levels of clinically relevant unrequested findings reported. Where an explanatory variable was not reported, we imputed it using simple median imputation (only relevant for the proportion of smokers).

## Results

### Systematic Review

The majority of the nineteen papers reviewed routine cardiac CT’s were conducted in convenience samples of patients with suspected Coronary Artery Disease (CAD) to determine the prevalence and significance of any unrequested findings. Three studies only retrospectively reviewed the radiology reports [19], [20], [21]; the prevalence of unrequested findings in these studies was not substantially different from that of studies prospectively evaluating the presence of unrequested findings. A number restricted their investigation to narrow cardio-centric FOV’s [22], [23], [24], [25] while others assessed reconstructions based on the maximally available FOV.

Several studies also drew a direct comparison between the unrequested findings detectable on full thoracic FOV and smaller, cardiac FOV. Kim et al. [26] compared the prevalence on LDCT scout views with a narrower cardiac-focused reconstructed FOV and found a very large discrepancy between the two, with the overwhelming majority of clinically relevant unrequested findings being missed in the narrower FOV. Aglan et al. [27] similarly compared the prevalence of unrequested findings observed with a narrow FOV with a full FOV using a split-sample approach. They also found far more unrequested finding on full ‘thoracic’ FOV. An indirect comparison between the prevalence’s reported in those articles based upon a restricted FOV and those based upon a full FOV did not show the same trend. This was confirmed quantitatively (see meta-analysis results below).

All studies distinguished between clinically relevant unrequested extra-cardiac findings and clinically irrelevant findings by classifying the former as those that require further action or follow-up and the latter as those that do not (non-relevant). Some also opted for a multimodal classification into mild, moderate and severe, with the latter two requiring some form of clinical action [19], [23], [28], [29].

This classification did not seem systematically pre-specified in any of the papers and was typically described pragmatically and briefly in the methods as based upon the attendant need for further follow-up or action according to the insights of the evaluating radiologists and cardiologists (with one exception, where raters simply filled in premade worksheets [26]). Some articles did explain how select, specific findings were handled, such as the criteria used to assess coronary artery aneurysms [30]. This is most notably the case for lung nodules, which two papers explicitly classified them according to the Fleischer [31] criteria [32], [29], whilst one paper [33] chose to classify all visible nodules as potentially relevant.

Four papers also reported whether the detected (potentially) relevant unrequested findings actually led to therapeutic or diagnostic consequences, chiefly through chart-review. Machaalany et al [28] found an overall prevalence of 8.2% of potentially relevant unrequested findings, of which 7% were indeterminate. They performed telephone and chart-review follow-up and found that no indeterminate findings had converted to relevant findings. Lehman et al. [34] investigated the number of unrequested findings observed in the course of an ongoing study conducted amongst patients presenting to their emergency room with acute chest pain. Whilst newly detected unrequested findings were detected in 20.5% of patients, patient management was only actually changed in 4.4% of cases overall. Similarly, Onuma et al. [35] found a prevalence of clinically relevant unrequested findings of 22.7% in patients suspected of CAD with 3.6% of the total population eventually having therapeutic consequences. In post CABG-patients, Mueller et al. [24] found 19.7% unrequested findings with documented follow-up in 9.6% of patients.

### Meta Analysis

**Figure 2:**
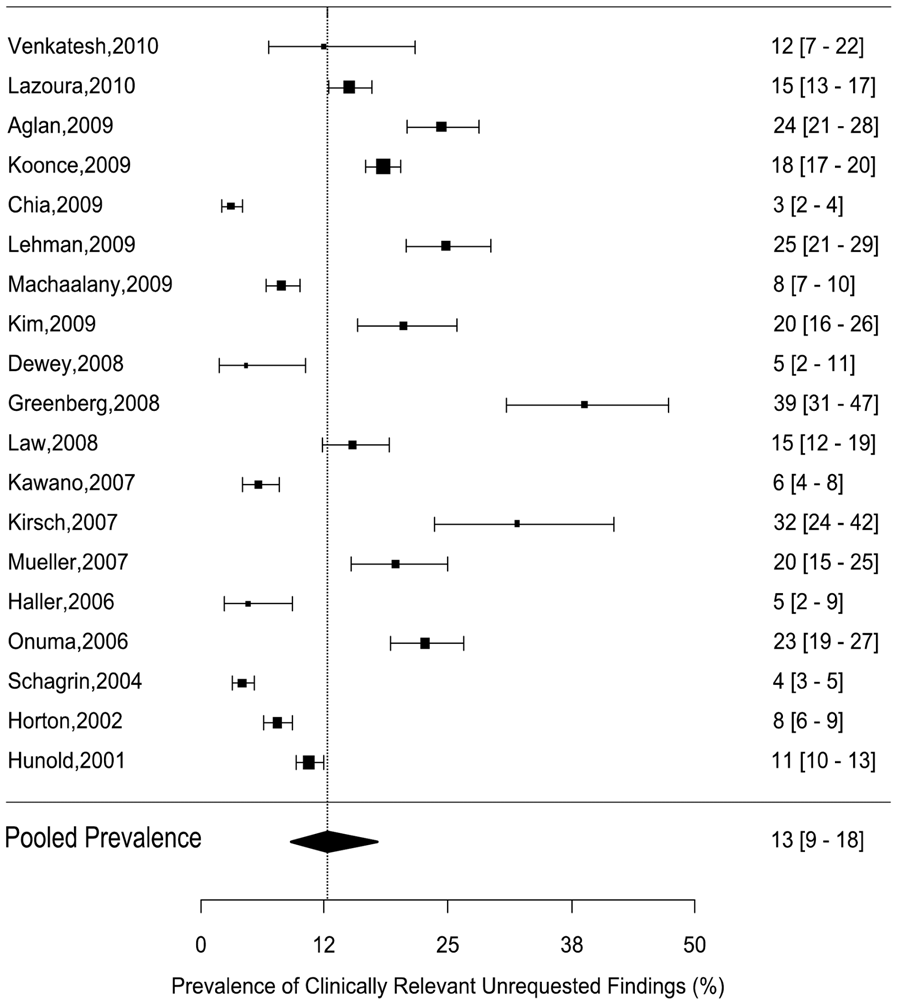
Forest plot of the included study showing the prevalence of clinically relevant unrequested findings and pooled prevalence estimate. The dotted line represents the pooled prevalence estimate, calculated using random effects. The estimated 95% confidence intervals are provided in brackets.

**Figure 3:**
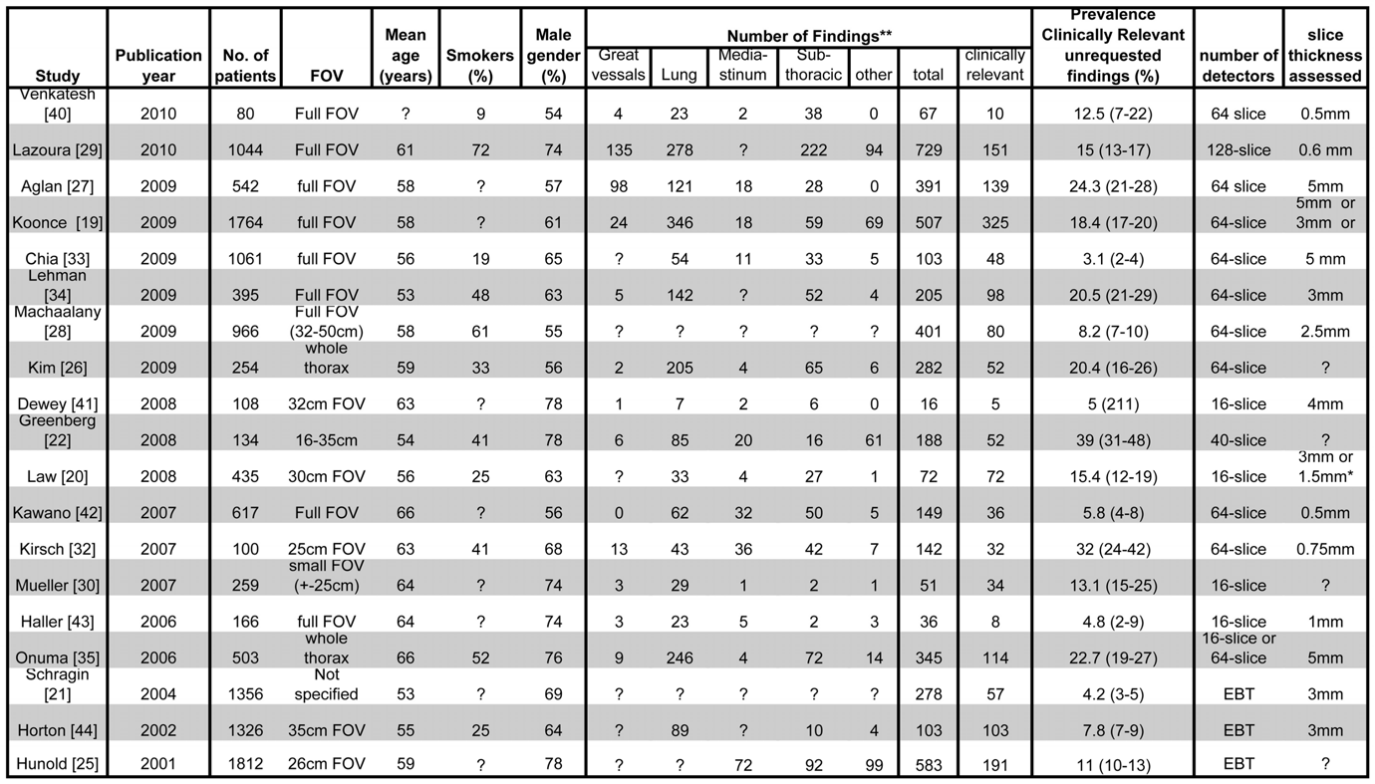
Overview of included articles with abstracted parameters. *depending on protocol used. EBT  =  Electron Beam Tomography. References for figure 3: Venkatesh [40], Lazoura [29], Aglan [27], Koonce [19], Chia [33], Lehman [34], Machaalany [28], Kim [26], Dewey [41], Greenberg [22], Law [20], Kawano [42], Kirsch [32], Mueller [30], Haller [43], Onuma [35], Schragin [21], Horton [44], Hunold [25].

The nineteen cardiac CT studies, incorporating 12922 patients, showed a pooled prevalence of clinically relevant unrequested findings of 13% (95% confidence interval: 9–18%, Figure 2). We found an overall I^2^ statistic of 98%. This suggests excess inter-study variability and correspondingly random effects were employed to generate pooled estimates. We found that the random-effects pooled prevalence estimates differed substantially from the fixed effects estimates, in keeping with the degree of heterogeneity suggested by the I2 (Figure 3). The funnel plot was approximately symmetrical, suggesting a low risk of publication bias (not reproduced here).

Univariate meta-regression for variables that could potentially explain this heterogeneity (Figure 3) did not yield any significant associations between the parameters assessed and the prevalence of clinically relevant unrequested findings in the dataset. We found no significance in mixed effects meta-regression for the proportion of smokers (p = 0.33), mean age of included subjects (p = 0.87), gender (p = 0.82), FOV (p = 0.59) and the number of years since publication (p = 0.26).

The STROBE items included and specified to our research question show the frequent absence of reporting of the referral source and the prevalence of other CVD risk-factors; only 37% of articles mention the proportions of study subjects included from different sources (i.e. primary care, specialist care, self-referral). We found that 14 articles mentioned cursory study patient characteristics but that these were usually limited to age and gender, with parameters such as smoking status missing in 8/19(42%) studies (not shown in table 2) and only 35% mentioning the cardinal CVD risk factors (smoking and hypertension and CVD history). We also observed that whilst all 19 studies reported numbers of unrequested findings (also an eligibility criterion); only 11% articles reported these data in such a way that the prevalence and absolute numbers of each (class of) unrequested finding could each be calculated. Many authors chose to report the absolute numbers of each finding in detail, but the possibility that single patients may have had multiple findings prevented accurate calculations of prevalence.

## Discussion

Unrequested findings were found to occur in approximately 13% of patients undergoing cardiac CT. This high overall prevalence is largely in line with what has been reported in screening settings [36].

Surprisingly, the high level of heterogeneity in prevalence on unrequested findings (i.e. 3–39%) was not explained by likely study and population characteristics, such as smoking and age. Similarly, imaging technique (i.e. FOV) did not explain the heterogeneity between studies. More detailed imaging and population characteristics that could have explained the heterogeneity were not systematically reported, as shown by the results of the STROBE quality check, with a only a third of articles fully describing the referral population source and reporting their risk profiles.

Differences in definition and classification of the endpoint, i.e. clinically relevant unrequested findings, is probably the largest contributor to the high level of heterogeneity. In each article the clinical relevance of the unrequested findings were defined based upon prevailing local insights and the expert opinion of the evaluating radiologists rather than any systematic evidence of prognostic significance, making it very difficult to begin to assess the nature of the criteria.

Consensus on the definition and classification of relevant unrequested findings is impossible in the absence of evidence concerning the prognostic and diagnostic value of such findings. Evidence supporting the wider prognostic value of (types of) unrequested findings might support more systematic reporting and acting-upon unrequested findings observed on cardiac CT and other scan-types by demonstrating their value and raising awareness. It is plausible that further research could also differentiate between findings with higher value and those with little or none. Such evidence would improve studies in this field, which until now have treated unrequested findings as large undifferentiated groups and assigned significance according to individual author’s insights.

The growing acceptance of more and earlier cardiovascular CT screening [37]–[39] amongst pre-symptomatic patients introduces further complication. Amongst these patients there is little precedent supporting the prognostic significance of unrequested findings in routine care settings. Whilst accurate risk stratification is more difficult amongst these patients, due to the longer time horizons and more subtle defects involved, the benefits of earlier targeted preventative measures might be correspondingly larger.

We found large discrepancies between the prevalence of clinically relevant (i.e. requiring follow-up) findings and the number of findings that actually led to therapeutic or diagnostic interventions [24], [28], [34], [35]. This suggests either a lack of communication between radiologists and clinicians, differences in the perceived clinical relevance of certain findings between these groups, or both. This seeming lack of consensus may have also contributed to the unexplained heterogeneity between the studies found in the meta-analysis.

### Limitations

We acknowledge that our study suffers from several limitations, including language limited to English, Dutch and German. Furthermore by choosing to limit our analysis to only cardiac CT, the prevalence’s we found may not be representative of the prevalence of unrequested findings in other anatomical regions or using other modalities. We examined the effects of study parameters on the prevalence of clinically relevant unrequested findings using the aggregate level data reported in the included studies and did not pursue the individual patient data (i.e. used mean patient age instead of the actual ages of all the individual patients). The latter approach is likely to have been more sensitive to subtle variations between the populations [25]. Furthermore, one of the parameters included in the meta-regression (smoking) was missing in almost half of the studies. Consequently we imputed this parameter using median imputation, further reducing its variability and hence the sensitivity of our analysis.

### Recommendations

In the absence of a standard definition of clinically relevant unrequested and to facilitate comparison between studies, we recommend that future studies transparently report the nature of the unrequested findings detail their absolute numbers and prevalence, the impact on patient care and outcome (if applicable), and the demographic and clinical characteristics of the source population. This is in lieu of reporting detailed criteria for clinical relevance, which may be impossible to pre-specify at this stage. In addition to adhering to the STROBE checklist, we suggest authors further specify the exact referral sources of patients included and report the prevalence of relevant risk factors, as specified in Table 2.

### Conclusion

We found a high prevalence of clinically relevant unrequested findings among published studies. The large range of prevalence’s could not be satisfactorily explained in this analysis. Further research to assess the true prognostic value of individual (sets of) unrequested findings that incorporates follow-up to measure associated patient outcomes would be desirable to inform an evidence-based response to the high prevalence of potentially clinically relevant unrequested information on thoracic CT scans.

## Supporting Information

Supplement S1 **MOOSE reporting checklist and location of items in article.**
(DOC)

## References

[1] Garvey CJ, Hanlon R (2002). Computed tomography in clinical practice.. BMJ.

[2] Scrutinio D, Giannuzzi P (2008). Comorbidity in patients undergoing coronary artery bypass graft surgery: impact on outcome and implications for cardiac rehabilitation.. Eur J Cardiovasc Prev Rehabil.

[3] Smith-Bindman R (2010). Is Computed Tomography Safe? N Engl J Med.. Jul 1;.

[4] Brenner DJ, Hricak H (2010). Radiation exposure from medical imaging: time to regulate?. JAMA.

[5] Hillman BJ, Goldsmith JC (2010). The Uncritical Use of High-Tech Medical Imaging. N Engl J Med.. Jul 1;.

[6] Mettler FA, Bhargavan M, Faulkner K, Gilley DB, Gray JE (2009). Radiologic and nuclear medicine studies in the United States and worldwide: frequency, radiation dose, and comparison with other radiation sources–1950–2007.. Radiology.

[7] Orme NM, Fletcher JG, Siddiki HA, Harmsen WS, O’Byrne MM (2010). Incidental findings in imaging research: evaluating incidence, benefit, and burden.. Arch Intern Med.

[8] Budoff MJ, Gopal A (2007). Incidental findings on cardiac computed tomography. Should we look?. J Cardiovasc Comput Tomogr.

[9] Sosnouski D, Bonsall RP, Mayer FB, Ravenel JG (2007). Extracardiac findings at cardiac CT: a practical approach.. J Thorac Imaging.

[10] Stroup DF, Berlin JA, Morton SC, Olkin I, Williamson GD (2000). Meta-analysis of observational studies in epidemiology: a proposal for reporting. Meta-analysis Of Observational Studies in Epidemiology (MOOSE) group. JAMA.. Apr 19;.

[11] Hussain A, Gordon-Dixon A, Almusawy H, Sinha P, Desai A (2009). The incidence and outcome of incidental breast lesions detected by computed tomography. Ann R Coll Surg Engl.. Mar;.

[12] Quentin M, Kropil P, Steiner S, Lanzman RS, Blondin D (2010). [Prevalence and clinical significance of incidental cardiac findings in non-ECG-gated chest CT scans.].. Radiologe Jan;.

[13] Vandenbroucke JP, von EE, Altman DG, Gotzsche PC, Mulrow CD (2007). Strengthening the Reporting of Observational Studies in Epidemiology (STROBE): explanation and elaboration.. Epidemiology.

[14] von EE, Altman DG, Egger M, Pocock SJ, Gotzsche PC (2008). The Strengthening the Reporting of Observational Studies in Epidemiology (STROBE) statement: guidelines for reporting observational studies.. J Clin Epidemiol.

[15] R Development Core Team (2011). http://www.R-project.org/.

[16] Viechtbauer W (2010). Conducting meta-analyses in R with the metafor package.. Journal of Statistical Software.

[17] Higgins JP, Thompson SG (2002). Quantifying heterogeneity in a meta-analysis.. Stat Med.

[18] DerSimonian R, Laird N (1986). Meta-analysis in clinical trials.. Control Clin Trials.

[19] Koonce J, Schoepf JU, Nguyen SA, Northam MC, Ravenel JG (2009). Extra-cardiac findings at cardiac CT: experience with 1,764 patients.. Eur Radiol.

[20] Law YM, Huang J, Chen K, Cheah FK, Chua T (2008). Prevalence of significant extracoronary findings on multislice CT coronary angiography examinations and coronary artery calcium scoring examinations.. J Med Imaging Radiat Oncol.

[21] Schragin JG, Weissfeld JL, Edmundowicz D, Strollo DC, Fuhrman CR (2004). Non-cardiac findings on coronary electron beam computed tomography scanning.. J Thorac Imaging.

[22] Greenberg-Wolff I, Uliel L, Goitein O, Shemesh J, Rozenman J (2008). Extra-cardiac findings on coronary computed tomography scanning.. Isr Med Assoc J.

[23] Kirsch J, Araoz PA, Steinberg FB, Fletcher JG, McCollough CH (2007). Prevalence and significance of incidental extracardiac findings at 64-multidetector coronary CTA.. J Thorac Imaging.

[24] Mueller J, Jeudy J, Poston R, White CS (2007). Cardiac CT angiography after coronary bypass surgery: prevalence of incidental findings.. AJR Am J Roentgenol.

[25] Hunold P, Schmermund A, Seibel RM, Gronemeyer DH, Erbel R (2001). Prevalence and clinical significance of accidental findings in electron-beam tomographic scans for coronary artery calcification.. Eur Heart J.

[26] Kim JW, Kang EY, Yong HS, Kim YK, Woo OH (2009). Incidental extracardiac findings at cardiac CT angiography: comparison of prevalence and clinical significance between precontrast low-dose whole thoracic scan and postcontrast retrospective ECG-gated cardiac scan.. Int J Cardiovasc Imaging.

[27] Aglan I, Jodocy D, Hiehs S, Soegner P, Frank R (2010). Clinical relevance and scope of accidental extracoronary findings in coronary computed tomography angiography: A cardiac versus thoracic FOV study. Eur J Radiol.. Apr.

[28] Machaalany J, Yam Y, Ruddy TD, Abraham A, Chen L (2009). Potential clinical and economic consequences of noncardiac incidental findings on cardiac computed tomography.. J Am Coll Cardiol.

[29] Lazoura O, Vassiou K, Kanavou T, Vlychou M, Arvanitis DL (2010). Incidental non-cardiac findings of a coronary angiography with a 128-slice multi-detector CT scanner: should we only concentrate on the heart?. Korean J Radiol.

[30] Mueller J, Jeudy J, Poston R, White CS (2007). Cardiac CT angiography after coronary bypass surgery: prevalence of incidental findings.. AJR Am J Roentgenol.

[31] MacMahon H, Austin JH, Gamsu G, Herold CJ, Jett JR (2005). Guidelines for management of small pulmonary nodules detected on CT scans: a statement from the Fleischner Society.. Radiology.

[32] Kirsch J, Araoz PA, Steinberg FB, Fletcher JG, McCollough CH (2007). Prevalence and significance of incidental extracardiac findings at 64-multidetector coronary CTA.. J Thorac Imaging.

[33] Chia PL, Kaw G, Wansaicheong G, Ho KT (2009). Prevalence of non-cardiac findings in a large series of patients undergoing cardiac multi-detector computed tomography scans.. Int J Cardiovasc Imaging.

[34] Lehman SJ, Abbara S, Cury RC, Nagurney JT, Hsu J (2009). Significance of cardiac computed tomography incidental findings in acute chest pain.. Am J Med.

[35] Onuma Y, Tanabe K, Nakazawa G, Aoki J, Nakajima H (2006). Noncardiac findings in cardiac imaging with multidetector computed tomography.. J Am Coll Cardiol.

[36] Burt JR, Iribarren C, Fair JM, Norton LC, Mahbouba M (2008). Incidental findings on cardiac multidetector row computed tomography among healthy older adults: prevalence and clinical correlates.. Arch Intern Med.

[37] Elias-Smale SE, Proenca RV, Koller MT, Kavousi M, van Rooij FJ (2010). Coronary calcium score improves classification of coronary heart disease risk in the elderly: the Rotterdam study.. J Am Coll Cardiol.

[38] Johnson KM, Dowe DA (2010). The detection of any coronary calcium outperforms Framingham risk score as a first step in screening for coronary atherosclerosis.. AJR Am J Roentgenol.

[39] Greenland P, Bonow RO, Brundage BH, Budoff MJ, Eisenberg MJ (2007). ACCF/AHA 2007 clinical expert consensus document on coronary artery calcium scoring by computed tomography in global cardiovascular risk assessment and in evaluation of patients with chest pain: A report of the American College of Cardiology Foundation Clinical Expert Consensus Task Force (ACCF/AHA Writing Committee to Update the 2000 Expert Consensus Document on Electron Beam Computed Tomography).. Circulation.

[40] Venkatesh V, You JJ, Landry DJ, Ellins ML, Sheth T (2010). Extracardiac findings in cardiac computed tomographic angiography in patients at low to intermediate risk for coronary artery disease.. Can Assoc Radiol J.

[41] Dewey M, Schnapauff D, Teige F, Hamm B (2007). Non-cardiac findings on coronary computed tomography and magnetic resonance imaging.. Eur Radiol.

[42] Kawano Y, Tamura A, Goto Y, Shinozaki K, Zaizen H (2007). Incidental detection of cancers and other non-cardiac abnormalities on coronary multislice computed tomography.. Am J Cardiol.

[43] Haller S, Kaiser C, Buser P, Bongartz G, Bremerich J (2006). Coronary artery imaging with contrast-enhanced MDCT: extracardiac findings.. AJR Am J Roentgenol.

[44] Horton KM, Post WS, Blumenthal RS, Fishman EK (2002). Prevalence of significant noncardiac findings on electron-beam computed tomography coronary artery calcium screening examinations.. Circulation.

