# Supplementary material for: Unrequested Findings on Cardiac Computed Tomography: Looking Beyond the Heart
Source: PLoS One. 2012 Apr 19;7(4):e32184. doi: 10.1371/journal.pone.0032184 (PMC3334960; doi:10.1371/journal.pone.0032184)
Supplement: Supplement S1 — MOOSE reporting checklist and location of items in article. (DOC) [file pone.0032184.s001.doc]

**MOOSE Statement - Reporting Checklist for Authors, Editors, and Reviewers of Meta-analyses of Observational Studies**

| **Reporting Criteria** | **Reported (Yes/No)** | **Reported on Page** |
| --- | --- | --- |
| **Reporting of Background** |  |  |
| Problem definition | YES | 3 |
| Hypothesis statement | N/A |  |
| Description of Study Outcome(s) | YES | 3 & 4 |
| Type of exposure or intervention used | YES | 3 |
| Type of study design used | YES | 3 |
| Study population | YES | 4 & Figure 1 |
| **Reporting of Search Strategy** |  |  |
| Qualifications of searchers (eg, librarians  and investigators) | YES | 4 |
| Search strategy, including time period  included in the synthesis and keywords | YES | 3 & Table 1 |
| Effort to include all available studies,  including contact with authors | NO |  |
| Databases and registries searched | YES | 4 & Table 1 |
| Search software used, name and  version, including special features used  (eg, explosion) | YES | 4 & Table 1 |
| Use of hand searching (eg, reference  lists of obtained articles) | YES | 4 |
| List of citations located and those  excluded, including justification | NO |  |
| Method for addressing articles  published in languages other than  English | YES | Figure 2 |
| Method of handling abstracts and  unpublished studies | NO |  |
| Description of any contact with authors | N/A |  |
| **Reporting of Methods** |  |  |
| Description of relevance or  appropriateness of studies assembled for  assessing the hypothesis to be tested | YES | 4 & Figure 2 |
| Rationale for the selection and coding of  data (eg, sound clinical principles or  convenience) | YES | 4 |
| Documentation of how data were  classified and coded (eg, multiple raters,  blinding, and interrater reliability) | YES | 4 |
| Assessment of confounding (eg,  comparability of cases and controls in  studies where appropriate | YES | Table 2 |
| Assessment of study quality, including  blinding of quality assessors;  stratification or regression on possible  predictors of study results YES 5 | YES | 5 & Table 2 |
| Assessment of heterogeneity | YES | 6 |
| Description of statistical methods (eg,  complete description of fixed or random  effects models, justification of whether  the chosen models account for predictors  of study results, dose-response models,  or cumulative meta-analysis) in sufficient  detail to be replicated | YES | 4 & 5 |
| Provision of appropriate tables and  graphics | YES | Table 3 and Figure 2 |
| **Reporting of Results** |  |  |
| Table giving descriptive information for  each study included | YES | Table 3 |
| Results of sensitivity testing (eg,  subgroup analysis) | N/A |  |
| Indication of statistical uncertainty of  findings | YES | 5 |
| **Reporting of Discussion** |  |  |
| Quantitative assessment of bias (eg,  publication bias) | YES | 5 |
| Justification for exclusion (eg, exclusion  of non–English-language citations) | YES | 4 & Figure 2 |
| Assessment of quality of included studies | YES | 6 & Table 2 |
| **Reporting of Conclusions** |  |  |
| Consideration of alternative explanations  for observed results | YES | 6 & 7 |
| Generalization of the conclusions (ie,  appropriate for the data presented and  within the domain of the literature review) | YES | 7 |
| Guidelines for future research | YES | 7, 8 & table 2 |
| Disclosure of funding source | YES | 8 |
